# Exposure to Multiple Low-Level Chemicals in Relation to Reproductive Hormones in Premenopausal Women Involved in Liquid Crystal Display Manufacture

**DOI:** 10.3390/ijerph10041406

**Published:** 2013-04-03

**Authors:** Ching-Chun Lin, Chia-Ni Huang, Jung-Der Wang, Yaw-Huei Hwang, Ruei-Hao Shie, Yu-Yin Chang, Shao-Ping Weng, Pau-Chung Chen

**Affiliations:** 1 Institute of Occupational Medicine and Industrial Hygiene, National Taiwan University College of Public Health, Taipei 100, Taiwan; E-Mails: ccleuven@gmail.com (C.-C.L.); jenny.huang@novartis.com (C.-N.H.); jdwang121@gmail.com (J.-D.W.); yhhwang@ntu.edu.tw (Y.-H.H.); rueihaoshie@itri.org.tw (R.-H.S.); yuyin.chang@gmail.com (Y.-Y.C.); shaopingw@gmail.com (S.-P.W.); 2 Department of Public Health, National Cheng Kung University College of Medicine, Tainan 701, Taiwan; 3 Green Energy and Environment Research Laboratories, Industrial Technology Research Institute, Hsinchu County 310, Taiwan; 4 Department of Public Health, National Taiwan University College of Public Health, Taipei 100, Taiwan; 5 Department of Environmental and Occupational Medicine, National Taiwan University College of Medicine and Hospital, Taipei 100, Taiwan

**Keywords:** estrogens, follicle stimulating hormone, liquid crystal display manufacturing, luteinizing hormone, premenopausal women, progesterone

## Abstract

*Background*: Liquid crystal display (LCD) manufacturing involves three fabrication processes: array, panel and module processes, which result in different levels of volatile organic compound (VOC) exposure. The aim of this study was to assess the potential reproductive endocrine effects of occupational exposures during LCD manufacturing predictive of menstrual cycles as subclinical markers of female reproductive dysfunction effects of low-dose exposures. *Methods*: A total of 94 fabrication workers were followed for one complete menstrual cycle using daily urine samples: 23 were from the array, 53 from the panel, and 18 from the module work areas. The menstrual cycle characteristics of the study population were measured using a self-administered questionnaire. Urine samples were collected during the first urination in the morning for at least one complete menstrual cycle. The urine was then analyzed to determine the urinary concentrations of follicular stimulating hormone (FSH), estrone conjugates (E1C), and pregnanediol-3-glucuronide (PdG). The results of this analysis were used to assess the potential effects of chemical exposure as determined by handheld volatile organic compound (VOC) monitors and 24 h canisters. *Results*: The concentration of total VOCs was much higher in the module making area (ND–21,000 ppb) than in panel (ND–766 ppb) and array (58–1,472 ppb) making areas. The concentrations of ethanol and acetone were much higher in the module (1,974.9 and 2,283.2 ppb, respectively) and panel (2256.9 and 592.2 ppb, respectively) making areas. Compared to those in the array making area, we found that E1C (12.55, 95% confidence interval (CI): 8.49, 16.61 μg/mg Cr) and PdG (0.53, 95% CI: 0.29, 0.77 μg/mg Cr) levels in the module group were significantly higher in the early follicular phase; E1C (11.93, 95% CI: 6.21, 17.65 μg/mg Cr) and PdG (0.53, 95% CI: 0.29, 0.77 μg/mg Cr) levels were significantly higher in the periovulatory phase; and all the hormone levels, FSH (1.48, 95% CI: 0.81, 2.15 μg/mg Cr), E1C (9.29, 95% CI: 4.92, 13.66 μg/mg Cr), and PdG (1.01, 95% CI: 0.42, 1.60 μg/mg Cr) were also significantly higher in the luteal phase. In addition, the FSH (0.89, 95% CI: 0.07, 1.71 μg/mg Cr) level in the panel group was significantly higher but E1C (−4.49, 95% CI: −7.90, −1.08 μg/mg Cr) was lower in the early follicular phase; and E1C (−5.16, 95% CI: −9.61, −0.71 μg/mg Cr) level was significantly lower in the periovulatory phase. *Conclusions*: Our findings add to the evidence that exposure to multiple low-level chemicals is associated with modest changes in reproductive hormone urinary concentrations in healthy premenopausal women. In addition, the FSH (0.89, 95% CI: 0.07, 1.71 μg/mg Cr) level in the panel group was significantly higher but E1C (−4.49, 95% CI: −7.90, −1.08 μg/mg Cr) lower in the early follicular phase; and E1C (−5.16, 95% CI: −9.61, −0.71 μg/mg Cr) level was significantly lower in the periovulatory phase.

## 1. Introduction

Liquid crystal display (LCD) manufacture is a rapidly growing, high-tech industry that uses many organic chemical solvents during the fabrication process. Few studies have discussed the effects of occupational exposure on menstrual cycle variability [1,2,3]. In semiconductor manufacturing, the supervising engineers and photolithography workers have a higher risk for longer menstrual cycles than non-fabrication workers. Workers with exposure to multiple chemicals, which includes ethyl glycol ethers (EGEs) in photolithography, have a greater risk of irregular menstrual cycles and endocrine disruption.

Many chemical agents with known reproductive toxicity are used in LCD manufacturing, such as propylene glycol monomethyl ether (PGME) [4], monoethanolamine (MEA) [5], *N*-methyl-2-pyrrolidinone (NMP) [6] and isopropanol [7]. Although their reproductive toxicity has been evaluated in animal studies, knowledge regarding their reproductive toxicity in humans is scarce. In our previous study [8], we found an increased risk of short menstrual cycles among women working in the panel and module areas. The hypothesis of our study was that the exposure of women to multiple organic solvents has substantial reproductive effects on premenopausal women. The potential reproductive toxicity in the LCD industry could result in the disruption of menstruation and infertility. This subsequent study used the daily urine metabolites of sex steroid hormones to obtain detailed menstrual function data and assess the potential reproductive effects of occupational exposure from LCD manufacturing.

The aim of this study was to assess the potential effects of occupational exposures by monitoring specific endocrine hormone levels in daily urination as a predictive measure of menstrual cycle activity and as subclinical biomarkers of reproductive health. Finally, the study will investigate the effect of organic solvents on the menstrual cycle characteristics of female workers in LCD manufacturing sites.

## 2. Material and Methods

### 2.1. Research Design and Population

This was a cross-sectional study that used urinary metabolites of sex steroids to explore the associations between menstrual function and occupational exposures of female employees at an LCD manufacturing plant in Taiwan. We explored the relationship between the hormone patterns of the workers to more easily characterize their menstrual cycles.

We conducted this cross-sectional survey during annual health examinations in December 2002 at an LCD manufacturing plant in Taiwan. This study included female workers with at least one year of employment at the plant, who completed a self-administered questionnaire of menstrual cycle characteristics and potential risk factors. The study population included premenopausal women aged 18–44 who were not pregnant or breast feeding, had not used oral contraceptives in the preceding year, had experienced menstrual cycles during that year, and did not have a history of treatment for menstrual irregularity, infertility, pelvic inflammatory diseases, endometriosis and/or major abdominal surgery.

A total of 178 workers were recruited for this study. The following workers were lost to follow-up: 63 subjects dropped out during the month of urine collection, 11 subjects were over 45 years old, two subjects had used hormone therapy during the study period, and three subjects did not record their menses in the diary. Of the initial 178 recruited subjects, only 94 workers could be analyzed. This study was approved by the National Taiwan University College of Public Health Ethic Review Board.

### 2.2. Processes of LCD Manufacturing

LCD manufacturing involves three major processes: array, panel (cell) and module production. The array process, which includes the steps of film deposition, photolithography, etching, and stripping, is similar to the semiconductor manufacturing process, except transistors are fabricated on a glass substrate and not on a silicon wafer. The panel or cell process includes the steps of printing, rubbing, liquid crystal injection, and joining the polarizer attachment to the arrayed back substrate and front substrate to fit with a color filter. The space between the two substrates is filled with liquid crystal during the module process, which includes the assembly, testing, and connection of additional components to the fabricated glass.

### 2.3. Data Collection

The baseline questionnaire collected information that included the subject’s age, working history, income group, marital status, education, lifestyle factors, an estimate of usual sleep duration, the number of previous pregnancies, and reproductive and menstrual histories. Lifestyle factors included occupational activity; sports activity; smoking, alcohol and caffeine consumption; and any special diet restrictions. Additionally, we collected information regarding the subjective symptoms of the visual, auditory, respiratory, cardiovascular, urinary, reproductive, digestive, blood, nervous, dermal, muscle and skeletal systems. Furthermore, we used the Chinese Health Questionnaire [9] for screening psychosocial stress. Based on validation studies of the Chinese Health Questionnaire, in which a score greater than or equal to four indicates overt psychiatric symptoms, we categorized females into low (0), middle (1–3), and high (≥4) stress levels.

The daily urine samples were collected for at least one menstrual cycle. All subjects were required to complete their menstrual diary, which include duration and cycle days, alcoholic consumption, coffee and caffeine consumption, second-hand smoke exposure, hours worked, shift time, hours slept, sexual intercourse, physical activity at home and work, and demographic information. The urine samples were collected at home and stored in freezer packs before being delivered to the laboratory where they were stored at −20 °C until the assays were perfomed.

Subjects marked their urine samples after the beginning of the first day of a menstrual cycle. If bleeding was reported, the subjects’ cycle starting dates were adjusted to 1 day earlier. The report of menses was accepted up to 14 days after data collection for participants with missing diary entries of menstrual bleeding

We estimated the day of ovulation by either the peak urinary concentration of estrone conjugates (E1C)/pregnanediol-3-glucuronide (PdG) or the peak urinary concentration of follicular stimulating hormone (FSH) and by the concurrence of the day of or the day after the E1C peak urinary concentration [10]. The menstrual cycle length was defined as the number of days from day 1 of menses to the day before day 1 of the next menses. The follicular phase length was defined as the number of days from day 1 of menses up to and including the day of the FSH surge. The luteal phase length was defined as the difference between the cycle length and the follicular phase. The definition of abnormal cycle lengths was an interval less than 24 or more than 35 days [11]. Cut-offs at 24 and 35 days were selected based on prospective studies showing that 10% of all cycles in premenopausal women were <24 days and 10% were >35 days [12]. Short luteal phase length was defined as 10 days or less. Long menses length was defined as 8 days or more [13].

### 2.4. Endocrine Measurements

The urine samples were analyzed for the estradiol metabolites estrone sulfate and estrone glucuronide (estrone conjugates [E1C]), the progesterone metabolite pregnanediol glucuronide (PdG) [14], and the total urinary FSH beta subunit [15]. We assayed the urinary metabolites of estrogen and progesterone with competitive enzyme immunoassays (EIA) and FSH with a triple-antibody enzyme-linked immunosorbent assay (ELISA). All of the protocols were approved by the laboratory of B.L. Lasley, University of California, Davis, CA, USA. Creatinine excretion was used to control for variation in the concentration of urine. FSH, E1C and PdG excretions were assessed during three different periods; the early follicular phase, defined as days 1–7 with day 1 as the first day of menstrual bleeding; the periovulatory phase, defined as ovulation ±3 days; and the luteal phase, defined as days 4–10 after the FSH surge to the day before the next menses.

### 2.5. Exposures Assessment

We divided the subjects into three fabrication groups (array, panel and module), and monitored their exposure to chemicals implicated in the literature as having possible reproductive toxicity. All exposure measurements were taken during normal operating conditions and should be representative of the usual working environment. Sampling dates were scheduled to avoid the annual maintenance period because of the increased probability of exposure to chemicals used for machine cleaning during this period.

We used hand-held volatile organic compound (VOC) monitors (PGM-4730, RAE System Inc., San Jose, CA, USA), each equipped with a photoionization detector (PID; range of detection, 1 ppb to 2,000 ppm), to determine total VOC concentration and distribution in the different fabrication areas. The monitors were used to sample total VOC concentrations in each working area by measuring each square meter during the subject’s working shift. These monitoring results therefore represent multiple measurements at different locations over a shift, which represents the workers’ exposure within each working area. To determine the concentrations of individual VOCs we also used 24 h canister collection in conjunction with gas chromatography/mass spectrometry (GC/MS) [9] in each fabrication area.

### 2.6. Statistical Analysis

We used the mixed linear regression model of the procedure “PROC MIXED” of the SAS statistical package (SAS Institute, Cary, NC, USA) to analyze the variation in FSH, E1C and PdG urinary concentrations in each phase of the menstrual cycle separately [16]. Age, body mass index (BMI), education, smoking, alcohol consumption, and working pattern were adjusted in the final models.

## 3. Results

We found that the ovarian steroid urinary concentrations could be related to adverse health outcomes. The VOCs and reproductive hazard ratings for office work and the three areas in fabrication are shown in Table 1. Photo ionization detection found that the level of atmospheric total VOC concentrations in the module area (21,000 ppb) was almost 14 times greater than in the array area (1,472 ppb) and 27 times greater than in the panel area (766 ppb).

Although the concentrations of individual organic compounds in the canister samples were well below the occupational exposure limits of the Taiwan Council of Labor Affairs, the concentrations of acetone and ethanol were the highest in the module and array areas, respectively.

A total of 94 fabrication workers contributed completed cycles of urine samples. Of the 94 women, 23 were from the array, 53 from the panel, and 18 from the module work areas. The array worker’s education was lower than the other groups. The work pattern of the module area workers required more 12 h shifts than the other work areas (Table 2). The participants’ ages, BMI, smoking, drinking, coffee consumption, and other working patterns showed no significant difference among the three work areas. The mean and standard deviation (SD) of the three major hormone groups in the three work areas during the three menstrual phases are shown in Table 3. 

**Table 1:** Air sampling levels of volatile organic compound with potential reproductive hazards by three areas of fabrication.

Volatile organic compounds (ppb) ^a^	Array	Panel	Module	Regulatory Standard (ppm)	Reproductive hazard ratings ^b^
Photo-ionization detector					
Range	58–1472	ND–766	ND–21,000		
Arenes and aromatics					
Toluene	20.8	7.5	11.1	100	A+
*m*/*p*-Xylene	6.9	1.7	1.1	100	A−
Benzene	1.1	0.5	0.7	5	A−
Ethylbenzene	4.6	0.6	0.7		B
Styrene	0.7	ND	0.1	50	A−
Alkynes					
Ethanol	281.6	2,256.9	1,974.9	1,000	A+
Acetone	58.9	592.2	2,283.2	750	A−
2-Butanone	4.5	2.9	3.5	200	A−
Isopropyl alcohol	23.8	4.3	81.0	400	B−
Ethyl acetate	2.6	1.8	2.0	400	A+
Methylene chloride	0.9	1.5	3.5	50	A+
Acetaldehyde	ND	0.6	1.0	100	A−
2-Methyl-1-propanol	ND	ND	46.5		B−
1-Butanol	30.1	ND	10.7	150	A−
Hexane	ND	ND	2.9	50	A−
3-Methylpentane,	ND	ND	2.1		E
2-Methylpentane,	1.8	ND	1.8		E
Methyl isobutyl ketone	0.3	ND	ND	50	E
1-Methoxy-2-propyl acetate	130.8	ND	ND		C
1-Methoxy-2-propanol,	36.9	ND	ND		B−

^a^ Analyzed by GC-MS from 24 h canister sampling; ^b^ Reproductive hazard ratings from Microdex (REPROTOX® system): A+, human reproductive hazard with no known no-effect dose; A, human reproductive hazard with known no-effect dose; A−, unconfirmed human reproductive hazard; B+, multiple reproductive effects in animals but no human data; B, mixed reproductive effects in animals but no human data; B−, few reproductive effects in animals but no human data; C, no reproductive data found; D, insufficient information to identify; E, known not to affect animal reproduction but no human data; and F, known not to affect human reproduction.

In the early follicular phase, we found that the FSH, E1C, and PdG urinary concentrations in the module group were higher than any other group. In the periovulatory phase, the module group also showed higher FSH and E1C urinary concentrations than any other work area groups. In the luteal phase, the E1C urinary concentrations in the panel group were lower than any other groups, but the FSH level in the module area was still higher than any other work area groups.

**Table 2:** Distributions of demographic and life style characteristics in female workers by three areas of fabrication.

Characteristics		Array	Panel	Module
		No. (%)	No. (%)	No. (%)
Number included		23 (100.0)	53 (100.0)	18 (100.0)
Age (years)				
	<30	12 (52.2)	22 (41.5)	4 (22.2)
	30–44	10 (43.5)	31 (58.5)	13 (72.2)
	≥45	1 (4.3)	0 (0.0)	1 (5.6)
Body mass index (kg/m^2^)				
	<18.5	7 (30.4)	11 (20.8)	4 (22.2)
	18.5–24.9	13 (56.5)	31 (58.5)	9 (50.0)
	≥25.0	3 (13.0)	11 (20.8)	5 (27.8)
Education (years)				
	<12	17 (73.9)	49 (92.5)	17 (94.4)
	≥12	6 (26.1)	4 (7.5)	1 (5.6)
Current smoking				
	Yes	2 (8.7)	5 (9.4)	1 (5.6)
	No	21 (91.3)	48 (90.6)	17 (94.4)
Current alcohol drinking				
	Yes	1 (4.3)	2 (3.8)	1 (5.6)
	No	22 (95.7)	51 (96.2)	17 (94.4)
Current coffee drinking				
	Yes	13 (56.6)	16 (30.2)	10 (55.6)
	No	10 (43.5)	37 (69.8)	8 (44.4)
Working pattern				
	Regular day work	20 (87.0)	44 (83.0)	12 (66.7)
	12 h shift work	3 (13.0)	9 (17.0)	6 (33.3)
Psychosocial score ^a^				
	Low (0)	17 (73.9)	44 (83.0)	17 (94.4)
	Middle (1–3)	6 (26.1)	9 (17.0)	0 (0.0)
	High (≥4)	0 (0.0)	0 (0.0)	1 (5.6)

^a^ Based on the Chinese Health Questionnaire [9].

**Table 3:** The mean concentrations of reproductive hormones by three areas of fabrication.

Reproductive hormones		Array		Panel		Module		*P* value ^a^
		Urine No.	Mean (SD)	Urine No.	Mean (SD)	Urine No.	Mean (SD)	
Early follicular phase ^b^								
	FSH (mIU/mg Cr)	156	0.51 (0.42)	393	0.56 (0.52)	148	0.65 (0.50)	0.005
	E1C (ng/mg Cr)	152	19.32 (18.20)	386	13.56 (12.17)	147	29.28 (30.35)	<0.001
	PdG (μg/mg Cr)	155	0.60 (0.87)	386	0.58 (0.92)	148	0.89 (1.51)	0.01
Periovulatory phase ^c^								
	FSH (mIU/mg Cr)	205	0.48 (0.53)	481	0.56 (0.78)	179	0.66 (1.49)	0.128
	E1C (ng/mg Cr)	205	43.17 (48.31)	474	35.04 (46.62)	179	50.08 (44.18)	0.001
	PdG (μg/mg Cr)	204	1.08 (1.20)	458	0.98 (1.29)	179	0.81 (1.09)	0.291
Luteal phase ^d^								
	FSH (mIU/mg Cr)	245	2.53 (2.58)	611	2.77 (3.03)	213	4.65 (5.66)	<0.001
	E1C (ng/mg Cr)	244	34.16 (24.53)	609	28.30 (22.18)	197	33.57 (29.17)	0.01
	PdG (μg/mg Cr)	245	2.23 (1.84)	608	2.26 (2.29)	218	2.75 (3.51)	0.20

Cr, creatinine; E1C, estrone conjugates; FSH, follicular stimulating hormone; PdG, pregnanediol-3- glucuronide; and SD, standard deviation. ^a^ Based on ANOVA tests. ^b^ Mean hormonal concentration at cycle day 1–7. ^c^ Mean hormonal concentration at day of ovulation ±3. ^d^ Mean hormonal concentration at day of ovulation +4–10.

Table 4 summarizes the crude and adjusted associations between work areas and reproductive hormones in each phase of the menstrual cycle. We found that E1C and PdG) urinary concentrations in the module group were significantly higher in the early follicular phase. Additionally, the E1C and PdG urinary concentrations were significantly higher in the periovulatory phase. 

**Table 4:** Associations between work areas and reproductive hormones in each phase of the menstrual cycle separately.

Reproductive hormones		Panel *vs.* array		Module *vs.* array		
		Crude	Adjusted ^a^	Crude	Adjusted ^a^	
		β (95% CI)	β (95% CI)	β (95% CI)	β (95% CI)	
Early follicular phase						
	FSH (mIU/mg Cr)	0.33 (−0.55, 1.21)	0.89 (0.07, 1.71) *	1.20 (0.12, 2.28) *	0.85 (−0.15, 1.85)	
	E1C (ng/mg Cr)	−5.77 (−9.10, −2.44) ***	−4.49 (−7.90, −1.08) **	9.95 (9.87, 10.03) ****	12.55 (8.49, 16.61) ****	
	PdG (μg/mg Cr)	0.03 (−0.17, 0.23)	0.004 (−0.19, 0.20)	0.32 (0.08, 0.56) **	0.53 (0.29, 0.77) ****	
Periovulatory phase						
	FSH (mIU/mg Cr)	0.74 (−0.63, 2.11)	1.06 (−0.23, 2.35)	1.72 (0.01, 3.43) *	1.02 (−0.59, 2.63)	
	E1C (ng/mg Cr)	−7.46 (−14.32, −0.60) *	−5.16 (−9.61, −0.71) *	7.40 (−1.22, 16.02)	11.93 (6.21, 17.65) ****	
	PdG (μg/mg Cr)	−0.03 (−0.23, 0.17)	0.004 (−0.20, 0.19)	0.32 (0.08, 0.56) **	0.53 (0.29, 0.77) ****	
Luteal phase						
	FSH (mIU/mg Cr)	0.25 (−0.32, 0.82)	0.20 (−0.35, 0.75)	2.21 (1.48, 2.94) ****	1.48 (0.81, 2.15) ****	
	E1C (ng/mg Cr)	−5.80 (−9.35, −2.25) ***	−3.44 (−6.99, 0.11)	0.59 (−3.88, 5.06)	9.29 (4.92, 13.66) ****	
	PdG (μg/mg Cr)	0.04 (−0.35, 0.43)	0.19 (−0.30, 0.68)	0.62 (0.13, 1.11) **	1.01 (0.42, 1.60) ***	

CI, confidence interval; Cr, creatinine; E1C, estrone conjugates; FSH, follicular stimulating hormone; and PdG, pregnanediol-3-glucuronide. ^a^ Adjusted for age, BMI, education, smoking, alcohol consumption, and working pattern. * < 0.05, ** < 0.01, *** < 0.001, **** < 0.0001.

The FSH, E1C, and PdG concentrations for the module group were also significantly higher in the luteal phase. In addition, the FSH urinary concentration in the panel group was significantly higher, but the E1C urinary concentration was lower in the early follicular phase, and the E1C urinary concentration was significantly lower in the periovulatory phase.

## 4. Discussion and Summary

The module group showed higher urinary E1C and PdG urinary concentrations in the early follicular and periovulatory phase and higher FSH, E1C and PdG urinary concentrations in the luteal phase. The concentration of total VOCs was much higher in the module manufacturing area than in other areas of the plant. In particular, the concentrations of ethanol and acetone were much higher in the panel- and module-making facilities.

In our previous study [8], we found that female employees working in the panel and module groups seemed to have a higher risk of shorter than normal menstrual periods. Particularly, the concentrations of ethanol and acetone in the panel and module areas were much higher than the other areas. Previous studies have shown that ethanol [17], acetone [18], toluene [19], xylene [20], benzene [21], and styrene [22] may affect female menstrual function. Although some toxic chemicals were also used in the array area, airborne concentrations were low or undetectable. Thus, multiple organic solvents, even in low-level concentrations, could cause menstrual cycle variations.

Some studies have found that ethanol could alter hormone urinary concentrations; even moderate alcohol consumption can alter hormone urinary concentrations and cause a reduction in the probability of conception [23,24]. The concentrations of ethanol in the module and panel areas were almost ten times higher than the other areas. Acetone has been suspected as an inhibitor of the function of the pituitary-ovarian axis. In human studies, three out of four female volunteers exposed to controlled low doses (1,000 ppm) of acetone for up to 7.5 h/day, 4 days/week had shorter than normal menstrual periods [25]. The repeated exposure to multiple chemicals can possibly cause irregular menstrual cycles even in low-dose exposures. The concentration of acetone in the module and the panel work areas was much higher than the array and non-fabrication areas (40× and 140×, respectively). The prevalence of alcohol drinking among females in Taiwan is only 3.4% [26]. In our study cohort, the prevalence of alcohol drinking was approximately 5%, consistent with the Taiwanese general population.

Previous studies have indicated that increasing urinary concentrations of FSH and E1C in the early follicular phase are an indication of reproductive aging, diminished ovarian oocyte reserve and ovarian failure [27]. An increase of FSH urinary concentration would reduce follicular phase length [28]. Throughout reproductive life, there is a steady reduction in the number of newly recruited ovarian follicles. During the follicular phase, there is a process of follicle recruitment, growth, and selection and synthesis of estrogens. Because FSH plays a central role in this process by stimulating the granulosa cells [29], a rise in FSH urinary concentration might affect follicular phase length, possibly by accelerating follicular development. Some studies have also suggested a different explanation stating that an earlier start of follicular growth may reduce the length of the follicular phase [30]. The follicular phase of the menstrual cycle shortens concomitantly with increasing FSH urinary concentrations. FSH stimulates estrogen production in granulosa cells; thus, the changes in estrogen (E1C) production are related to increased FSH.

However, some unexpected results were found in the panel group. This includes lower E1C urinary concentrations in the early follicular and periovulatory phase and higher FSH urinary concentration in the early follicular phase. The reasons for the discrepancies between the panel and model groups are unclear. Although the concentrations of ethanol and acetone by 24 h canister collection were similar between the panel and module areas, the concentration of total VOCs was much lower in the panel area than in module area. Moreover, one might speculate that other reproductive toxicants may exist in the environment. Thus, further studies using individual exposure assessment are required to elucidate the causal relationship in the panel group.

Our study has some potential limitations. Selection bias could have resulted if the women chosen to participate had both menstrual problems and related reproductive outcomes, such as infertility, while they experienced the exposure of interest. However, we restricted our analysis to those who completed the questionnaires and met a strict set of reproductive criteria. Menstrual cycle characteristics were assessed using questionnaires as part of their annual health examination. We omitted women who were currently breast feeding, pregnant, taking oral contraceptives, or had recently ended a pregnancy. These women were omitted because they may have had cycles that did not reflect their typical pattern. Oral contraceptives are often prescribed for women who are having menstrual irregularities; therefore, our data may underestimate the prevalence of irregular cycles in our population. Furthermore, there could be a healthy worker effect because workers with ailments were likely to miss the annual health examination or to quit their jobs. Conversely, since female workers might quit their jobs because of pregnancy while those who are infertile remain at work there could be an underestimation of the risk from exposure. 

Our findings add evidence that exposure to multiple low-level chemicals is associated with modest changes in reproductive hormone urinary concentrations in healthy premenopausal women. The module work area is exposed to low concentrations of chemicals, but the multiple chemical exposures may diminish ovarian oocyte reserves or induce ovarian failure. Shortening of the follicular phase leads to shortening of the menstrual cycle. Further research of individual exposure assessment is warranted to assess the potential reproductive effects from occupational exposure to volatile organic compounds.
